# A NanoLuc-Based Protease Biosensor for Highly Sensitive Detection of Intracellular Protease Activity: Applications to Apoptosis and Coronavirus Infection

**DOI:** 10.21769/BioProtoc.5833

**Published:** 2026-09-20

**Authors:** Masashi Arakawa, Eiji Morita

**Affiliations:** Department of Biochemistry and Molecular Biology, Faculty of Agriculture and Life Science, Hirosaki University, Hirosaki, Aomori, Japan

**Keywords:** FlipNanoLuc, Protease sensor, Coronavirus, SARS-CoV-2, 3CL protease, NanoLuc, Apoptosis, Protease activity reporter

## Abstract

FlipNanoLuc is a highly sensitive protease biosensor based on the β-strand-flipping principle of NanoLuc luciferase, which is derived from *Oplophorus gracilirostris*. In the inactive configuration, one β-strand of NanoLuc is repositioned, thereby suppressing luciferase activity. Upon cleavage of the embedded protease recognition sequence by a target protease, the flipped β-strand is released, and luciferase activity is reconstituted. Incorporation of the LgBiT fragment (NanoBiT technology) yields strong luminescent output once the reporter is reconstituted, whereas the CL1-PEST1 degradation tag lowers background luminescence by promoting the degradation of the uncleaved, inactive form; together, these two modifications widen the dynamic range. A firefly luciferase normalization cassette connected via a P2A self-cleaving peptide is encoded in the same reporter plasmid, thereby eliminating the need for separate co-transfection. Because the readout directly reports intracellular protease activity in living cells, the system is suitable for detecting protease activation during apoptosis or viral infection and, in principle, for evaluating protease inhibitors and antiviral compounds. This protocol describes the following: (1) generation of HEK293T cells stably expressing FlipNanoLuc by retroviral transduction; (2) validation of reporter activity by protease overexpression; (3) detection of apoptosis using staurosporine; (4) detection of human coronavirus OC43 infection; and (5) detection of SARS-CoV-2 infection in BHK cells stably expressing hACE2. All luminescence assays employ dual-luciferase normalization and are compatible with standard 384-well plate readers.

Key features

• Highly sensitive detection of intracellular protease activity using a β-strand-flipping NanoLuc biosensor with dual-luciferase normalization encoded in a single plasmid.

• Detects protease activity in living cells, including caspase-3 during apoptosis and 3CLpro during authentic OC43 and SARS-CoV-2 infection.

• Quantitative luminescence readout in a 384-well plate format with a standard plate reader.

• Adaptable to other proteases and to inhibitor or antiviral screening by exchanging the embedded recognition sequence.

## Graphical overview


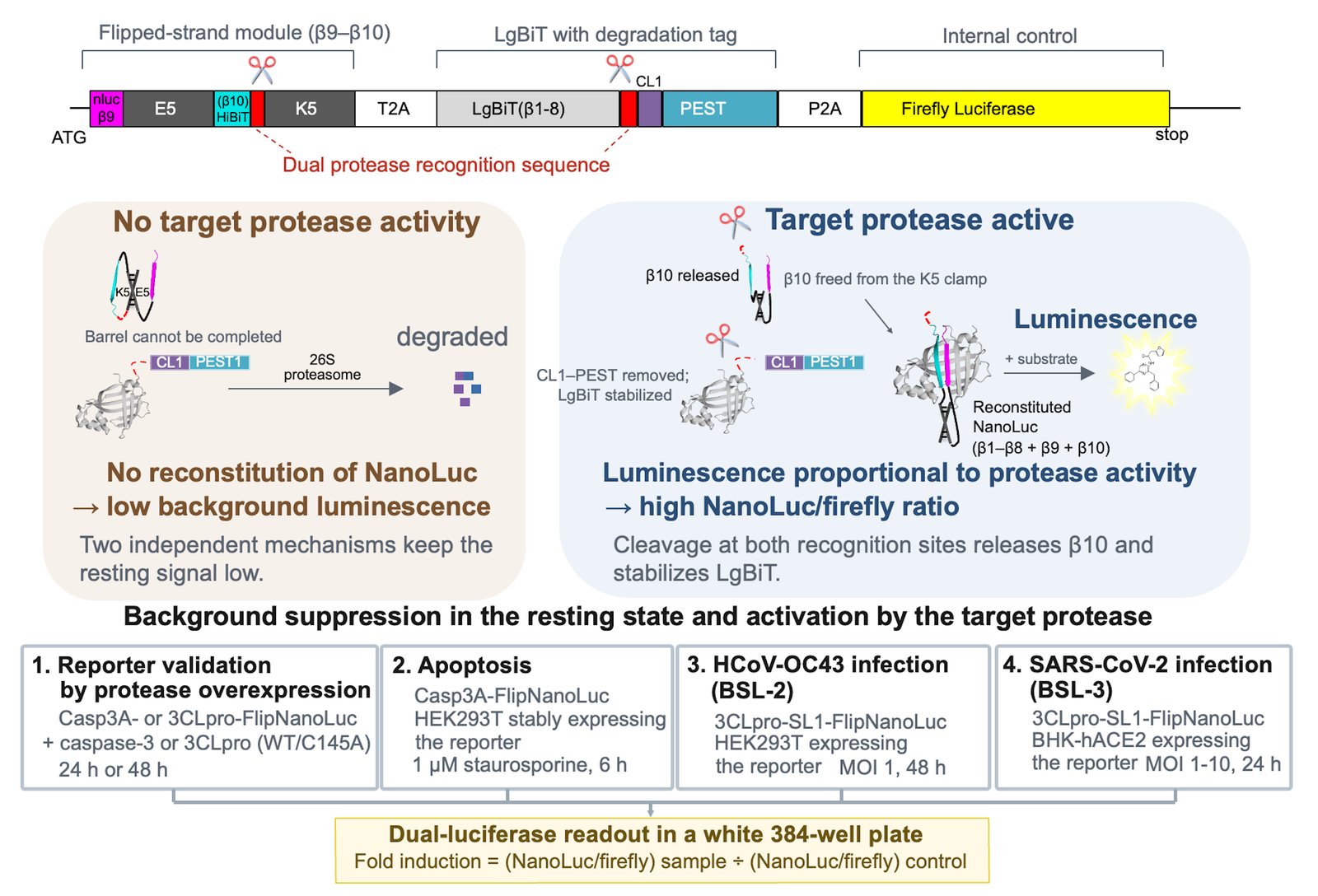


**Principle and applications of the NanoLuc-based protease biosensor FlipNanoLuc.** Architecture of the reporter, which is encoded as a single transcript (top panel). Suppression of the resting signal and activation of the reporter by the target protease (middle panels). The four applications described in this protocol are all read out by dual-luciferase measurement in a 384-well format (bottom panels).

## Background

Protease activity is a central regulatory event in diverse biological processes, including apoptosis, viral replication, and innate immune signaling. Therefore, sensitive and quantitative detection of protease activity in living cells is of broad utility in both basic research and antiviral drug discovery.

Bioluminescent reporters based on NanoLuc luciferase (NLuc) offer high sensitivity and a wide dynamic range compared with conventional firefly or Renilla luciferase systems. The NanoBiT complementation system [1] splits NLuc into a large fragment (LgBiT, 18 kDa) and a small peptide (SmBiT or HiBiT), thereby enabling the reconstitution-based detection of protein–protein interactions. Splitting NLuc in this way is advantageous for the design of activity reporters: the separated fragments are essentially non-luminescent on their own, so reconstitution can be made strictly dependent on a designed molecular event, whereas the high affinity of the LgBiT–HiBiT pair restores strong luminescence once reconstitution is permitted. Because HiBiT is a short peptide, it can also be repositioned within an engineered polypeptide with little steric penalty, which is not feasible with intact NLuc or with the substantially larger firefly and Renilla luciferases [1,2].

FlipNanoLuc is encoded as a single transcript that yields three products: a flipped-strand module, the LgBiT fragment of NLuc carrying a CL1-PEST1 destabilization tag, and firefly luciferase, which are separated by T2A and P2A self-cleaving peptides (Graphical overview, top panel). In the flipped-strand module, the β10 strand of NLuc (HiBiT) is held by an E5/K5 coiled-coil pair in a flipped, parallel orientation relative to β9, so that the NLuc barrel cannot be completed, and catalytic activity is abolished. A protease recognition sequence is placed within the flexible linkers of this module and of the LgBiT fragment. Cleavage by the target protease releases β10 from the coiled-coil constraint and removes the CL1-PEST1 tag from LgBiT, allowing the strand to re-engage the barrel, reconstituting luminescence (Graphical overview, middle panels). Conversely, in the absence of protease activity, the reporter remains suppressed by two independent mechanisms: LgBiT requires HiBiT for full activity, and the CL1-PEST1 tag accelerates the degradation of the uncleaved form before it can fold spontaneously. Together, these features yield a reporter with a low baseline signal and high fold induction upon protease activation [3].

Because reporter cleavage depends on the catalytic activity of the target protease, FlipNanoLuc provides a quantitative, live-cell activity readout that extends beyond simple detection. The embedded recognition sequence is modular and can be exchanged to monitor other cellular or viral proteases. The same readout can, in principle, be applied to the evaluation of protease inhibitors and antiviral compounds, as the inhibition of the target protease is reflected by reduced reporter induction (for example, 3CLpro inhibition during coronavirus infection).

This protocol details the complete workflow, from stable cell line generation to luminescence data acquisition and normalization, covering four experimental applications: protease overexpression validation, staurosporine-induced apoptosis, OC43 coronavirus infection, and SARS-CoV-2 infection.

## Materials and reagents

**Biological materials**

1. HEK293T cells (ATCC, catalog number: CRL-3216)

2. BHK cells stably expressing hACE2 (parental BHK cells; JCRB Cell Bank, catalog number: JCRB9020); generated in-house using the PiggyBac transposon system; see General notes

3. SARS-CoV-2 (BSL-3); the virus stock is the same as that described in Yoshida et al. [4] and must be obtained under appropriate institutional and regulatory approval

4. Human coronavirus OC43 (BSL-2) (ATCC, catalog number: VR-1558)

5. FlipNanoLuc reporter plasmids (available via Addgene; see Table 1 for the plasmid list)

**Table 1.** FlipNanoLuc reporter and protease expression plasmids used in this protocol, with their Addgene accession numbers.

Construct (used in this protocol)	Addgene plasmid name	Addgene ID
3CLpro-FlipNanoLuc reporter, CoVA WT	pQC.Flip-nluc(LgBiT1-8)CP[CoVA]-Fluc	200125
3CLpro-SL1-FlipNanoLuc reporter, CoVA WT (used for SARS-CoV-2/OC43 infection assays)	pQC.SARS2_5′UTR(SL1)-Flip-nluc(LgBiT1-8)CP[CoVA]-Fluc	200126
3CLpro-SL1-FlipNanoLuc reporter, CoVA Q5A (cleavage-site mutant, negative control)	pQC.SARS2_5′UTR(SL1)-Flip-nluc(LgBiT1-8)CP[CoVA(Q5A)]-Fluc	200127
3CLpro-SL1-FlipNanoLuc reporter, CoVA SA (cleavage-site mutant, negative control)	pQC.SARS2_5′UTR(SL1)-Flip-nluc(LgBiT1-8)CP[CoVA(SA)]-Fluc	200128
Casp3A-FlipNanoLuc reporter, WT (apoptosis assay)	pQC.Flip-nluc(LgBiT1-8)CP[Casp3A]-Fluc	200123
Casp3A-FlipNanoLuc reporter, D5A (cleavage-site mutant, negative control)	pQC.Flip-nluc(LgBiT1-8)CP[Casp3A(D5A)]-Fluc	200124
Caspase-3 (WT) protease expression plasmid (apoptosis/reporter validation)	pCAG.CASP3-Myc	200119

*Note: Construct names follow the Addgene depositor records. CoVA Q5A and SA, and Casp3A D5A, are cleavage-site mutants used as negative controls. pCAG.CASP3-Myc encodes the caspase-3 protease (not a FlipNanoLuc reporter) and is used to validate the Casp3A reporter. The Q5A and SA cleavage-site mutants are available only in the SL1-containing backbone; see the note in Part 2, section B.*

6. Gag-pol expression plasmid for retroviral particle production, as described in Morita et al. [5]

7. VSV-G expression plasmid for retroviral particle production, as described in Naldini et al. [6]

8. Protease expression plasmids: SARS-CoV-2 3CLpro, wild-type (pCAG.Myc-SARS2_3CL; Addgene, plasmid number: 200120) and the catalytically inactive C145A mutant [pCAG.Myc-SARS2_3CL(C145A); Addgene, plasmid number: 200121]; caspase-3, wild-type (pCAG.CASP3-Myc; Addgene, plasmid number: 200119)

9. PiggyBac transposon vector encoding hACE2, constructed in PB-CMV-MCS-EF1α-RedPuro (System Biosciences, catalog number: PB514B-2)

10. Super PiggyBac transposase expression vector (System Biosciences, catalog number: PB200A-1; currently supplied as catalog number: PB210PA-1)

11. Empty vector (pCAG-empty), constructed in-house; the pCAG backbone without an insert was used as the mock control in Tables 2 and 3

**Table 2.** Plasmid combinations for the 3CLpro (SARS-CoV-2) overexpression assay. In every condition, 0.5 μg of reporter plasmid and 0.5 μg of co-transfected plasmid are used per well (1:1, w/w; 1 μg of total DNA).

Condition	Reporter plasmid	Reporter Addgene ID	Co-transfected plasmid	Purpose/expected result
Test	3CLpro-FlipNanoLuc (CoVA WT)	200125	SARS-CoV-2 3CLpro (WT)	Active protease cleaves the reporter → high NanoLuc signal
Protease-inactive control	3CLpro-FlipNanoLuc (CoVA WT)	200125	SARS-CoV-2 3CLpro (C145A)	Catalytically dead protease → no signal; confirms dependence on protease activity
Cleavage-site specificity reference	3CLpro-SL1-FlipNanoLuc (CoVA WT)	200126	SARS-CoV-2 3CLpro (WT)	Reference for the cleavage-site mutant control below
Reporter cleavage-site mutant control	3CLpro-SL1-FlipNanoLuc (CoVA Q5A or SA)	200127 or 200128	SARS-CoV-2 3CLpro (WT)	Non-cleavable recognition site → no signal; confirms sequence specificity
Mock	3CLpro-FlipNanoLuc (CoVA WT)	200125	Empty vector (pCAG-empty)	No protease → background reference

*Note: The Q5A and SA cleavage-site mutants exist only in the SL1-containing backbone. For cleavage-site specificity experiments, therefore, use the SL1-containing WT reporter (number: 200126) as the reference and compare it with the SL1-containing Q5A and SA reporters (numbers: 200127 and 200128), so that the constructs differ only in the protease recognition sequence. Do not compare the mutant reporters directly with the reporter used in the other conditions (number: 200125), which lacks SL1, because the two constructs differ in their 5′UTR and therefore in the level of reporter expression.*

**Table 3.** Plasmid combinations for the caspase-3 (apoptosis) overexpression assay. In every condition, 0.5 μg of reporter plasmid and 0.5 μg of co-transfected plasmid are used per well (1:1, w/w; 1 μg of total DNA).

Condition	Reporter plasmid	Reporter Addgene ID	Co-transfected plasmid	Purpose/expected result
Test	Casp3A-FlipNanoLuc (WT)	200123	Caspase-3 (WT)	Active caspase-3 cleaves the reporter → high NanoLuc signal
Reporter cleavage-site mutant control	Casp3A-FlipNanoLuc (D5A)	200124	Caspase-3 (WT)	Non-cleavable recognition site → no signal; confirms sequence specificity
Mock	Casp3A-FlipNanoLuc (WT)	200123	Empty vector (pCAG-empty)	No protease → background reference

**Cell culture reagents**

1. Dulbecco's modified Eagle medium (DMEM), high glucose (Nacalai Tesque, catalog number: 08458-16)

2. Minimum essential medium (MEM) (Sigma-Aldrich, catalog number: M4655)

3. Fetal bovine serum (FBS) (Gibco, Thermo Fisher Scientific, catalog number: 10270-106); heat-inactivate before use (see Recipes)

*Note: This catalog number is currently listed as unavailable by the supplier; an equivalent qualified FBS may be used.*

4. Benzylpenicillin potassium (Fujifilm Wako Pure Chemical Corporation, catalog number: 021-07732)

5. Streptomycin sulfate (Tokyo Chemical Industry, catalog number: S0585)

6. Puromycin dihydrochloride (InvivoGen, catalog number: ant-pr-1)

7. Opti-MEM I reduced serum medium (Thermo Fisher Scientific, catalog number: 31985070)

8. Phosphate-buffered saline (PBS), without calcium and magnesium (Nacalai Tesque, catalog number: 14249-24)

9. Trypsin-EDTA solution (0.25%) (Nacalai Tesque, catalog number: 32777-15)

**Transfection reagents**

1. Polyethylenimine (PEI MAX, MW 40,000; 1 mg/mL stock in water) (Polysciences, catalog number: 24765-1)

**Luminescence assay reagents**

1. Nano-Glo^®^ luciferase assay system (Promega, catalog number: N1120)

2. Bright-Glo^TM^ luciferase assay system (Promega, catalog number: E2620)

**Lysis buffer components**

1. NaCl (Nacalai Tesque, catalog number: 31320-05)

2. Tris (Tris[hydroxymethyl]aminomethane) (Nacalai Tesque, catalog number: 35406-91)

3. Triton X-100 (Nacalai Tesque, catalog number: 35501-15)

4. cOmplete^TM^ Mini protease inhibitor cocktail tablets (Roche, catalog number: 11836153001)

5. Hydrochloric acid (HCl), for pH adjustment

6. Distilled water (used for the preparation of buffers and stock solutions)

**Apoptosis assay**

1. Staurosporine (AdipoGen Life Sciences, catalog number: AG-CN2-0022; CAS 62996-74-1); prepare 200 μM stock in DMSO

2. Dimethyl sulfoxide (DMSO) (Nacalai Tesque, catalog number: 13406-55)

**Solutions**

1. 1× lysis buffer (see Recipes)

2. 100× penicillin G + streptomycin stock solution (see Recipes)

3. Complete culture medium (see Recipes)

**Recipes**

**1. 1× lysis buffer**

**Table:** 

Component	Final concentration
NaCl	150 mM
Tris-HCl, pH 7.4	50 mM
Triton X-100	1% (v/v)
Protease inhibitor cocktail	1×

Dissolve NaCl and Tris-HCl in approximately 80% of the final volume of distilled water. Add Triton X-100 and mix until dissolved. Adjust the pH to 7.4 using HCl. Bring to the final volume. Store at 4 °C for up to one month.

*Note: Add the protease inhibitor cocktail immediately before use, at one tablet per 10 mL of lysis buffer (1×).*

**2. 100× penicillin G + streptomycin stock solution**

Dissolve 0.626 g of benzylpenicillin potassium and 1 g of streptomycin sulfate in PBS to a final volume of 100 mL. Sterilize through a 0.22 μm filter and store at 4 °C for up to 6 months; for longer storage, dispense into aliquots and keep at -20 °C. This stock is used at 1× (1:100 dilution) in the respective complete culture medium (DMEM for HEK293T cells and MEM for BHK cells stably expressing hACE2).

*Note: In this protocol, Pen/Strep refers to the homemade 100× penicillin G + streptomycin stock solution described in this recipe. A commercial penicillin-streptomycin solution may be substituted at the manufacturer’s recommended working concentration.*

**3. Complete culture medium**

Combine 500 mL of basal medium (DMEM for HEK293T cells and MEM for BHK cells stably expressing hACE2), 50 mL (final 10%) of FBS that has been heat-inactivated (56 °C, 45 min), and 5 mL (final 1×) of 100× penicillin G + streptomycin stock solution. Mix gently. Store at 4 °C for up to 2 months. For the maintenance medium of BHK cells stably expressing hACE2, additionally supplement with puromycin dihydrochloride to a final concentration of 3 μg/mL.

**Laboratory supplies**

1. White 384-well plates (Greiner Bio-One, catalog number: 784075)

2. 24-well cell culture plates (Violamo, catalog number: 2-8588-03)

3. 96-well cell culture plates (Violamo, catalog number: 2-8588-05)

4. 3.5 cm (35 mm) cell culture dishes (Thermo Scientific, catalog number: 130180)

5. Microcentrifuge tubes, 1.5 mL (Watson, catalog number: 131-815C)

6. Syringe filters, 0.22 μm (AS ONE, catalog number: 033022SO-SFCA)

## Equipment

1. Varioskan LUX multimode plate reader (Thermo Fisher Scientific, catalog number: VLBL00D1) or an equivalent luminometer capable of luminescence detection in 384-well format

2. Multichannel pipette (suitable for 10 μL) (Thermo Scientific, catalog number: 4661040N)

3. Refrigerated microcentrifuge capable of 500× *g* at 4 °C (Thermo Scientific, catalog number: 75002559)

4. BSL-2 and BSL-3 certified biosafety cabinets (as appropriate) (HITACHI, catalog number: SCV-1303ECIIB)

5. 37 °C incubator with 5% CO_2_ (PHC, catalog number: MCO-170AIC-PJ)

6. Inverted phase-contrast microscope (OLYMPUS, model: CKX53)

7. Water bath capable of 56 °C (Yamato Scientific, catalog number: BF200)

## Software and datasets

1. GraphPad Prism (version 11.0.0 for macOS) (GraphPad Software, https://www.graphpad.com)

2. SkanIt Software for microplate readers (Thermo Fisher Scientific), used for data acquisition on the Varioskan LUX plate reader v. 6.0.2.3

## Procedure

**Part 1: Generation of cells stably expressing FlipNanoLuc by retroviral transduction**

**A. Preparation of retroviral particles in HEK293T packaging cells**

1. One day before transfection, seed HEK293T cells in a 3.5 cm dish at a density of 5 × 10^5^ cells/dish in 2 mL of complete DMEM.

2. Incubate overnight at 37 °C with 5% CO_2_.

3. On the day of transfection, confirm by phase-contrast microscopy that cells are healthy (well attached, of normal morphology, and with few rounded or floating cells) and approximately 70%–80% confluent. Do not replace the medium at this point; the DNA–PEI complex is added directly to the existing culture medium.

4. Prepare the following plasmid mixture in 200 μL of Opti-MEM:

a. FlipNanoLuc reporter plasmid (pQCxIP-based retroviral vector): 1.0 μg

b. Gag-pol expression plasmid: 0.7 μg

c. VSV-G expression plasmid: 0.3 μg

d. Total: 2.0 μg

5. Vortex briefly. Add 6 μL of 1 mg/mL PEI MAX (DNA:PEI ratio = 1:3, w/w). Vortex briefly.

6. Incubate at room temperature (20–25 °C) for 15–20 min.

7. Add the DNA–PEI complex dropwise to the cells, then gently rock the dish by hand (approximately 10 times in each of the forward–backward and side-to-side directions) to distribute the complex evenly.

8. Incubate at 37 °C with 5% CO_2_ for 48 h without medium replacement.

*Notes:*

*1. VSV-G pseudotyped retroviral particles have a broad host range and should be handled with appropriate biosafety precautions.*

*2. Transfection is carried out in complete DMEM containing Pen/Strep. In our experience, the presence of these antibiotics does not compromise the efficiency of PEI-mediated transfection; however, this has not been systematically examined.*

**B. Harvest of retroviral supernatant**

1. At 48 h post-transfection, collect approximately 1 mL of the culture supernatant into a 1.5 mL microcentrifuge tube.

2. Centrifuge at 500× *g* for 5 min at 4 °C to remove cell debris.

3. Transfer the clarified supernatant to a new 1.5 mL microcentrifuge tube.

4. Use immediately or store at -80 °C.

*Notes:*

*1. Avoid repeated freeze/thaw cycles. Aliquot before freezing.*

*2. The retroviral titer is not determined in this protocol. Viral input is instead standardized by preparing every batch under identical conditions (the same number of packaging cells, the same plasmid amounts and ratio, and harvest at 48 h post-transfection) and by using a fixed volume of clarified supernatant (200 μL per 3.5 cm dish) for transduction. If the transduction efficiency varies between preparations, determine the titer of the supernatant (for example, by counting puromycin-resistant colonies after limiting dilution) or adjust the input volume accordingly.*

**C. Seeding of target HEK293T cells for transduction**

1. One day before transduction, seed HEK293T cells in a 3.5 cm dish at 1 × 10^5^ cells/dish in 2 mL of complete DMEM.

2. Incubate overnight at 37 °C with 5% CO_2_.

**D. Retroviral transduction**

1. Aspirate the culture medium from target cells.

2. Add 200 μL of clarified retroviral supernatant to the cells.

3. Add complete DMEM to the 3.5 cm dish to bring the total volume to 2 mL.

4. Incubate at 37 °C with 5% CO_2_ for 48 h.

**E. Puromycin selection**

1. At 48 h post-transduction, replace the medium with 2 mL per 3.5 cm dish of complete DMEM containing 1.0 μg/mL puromycin.

2. Replace the selection medium every 2–3 days.

3. Continue until all cells in a non-transduced control dish have died (typically 3–5 days).

4. Expand surviving cells as a pooled stable population.

*Notes:*

*1. Always include a non-transduced control dish under puromycin selection in parallel. One control dish per selection batch is sufficient because it serves only to define the endpoint of selection and does not need to be matched one-to-one with the experimental dishes.*

*2. Single-cell cloning is not required; pooled stable populations are suitable for downstream assays.*

*3. Confirm that the stable population has been established before use. Two measurements are used in this protocol: (i) functional confirmation, in which the stable cells are transfected with the corresponding protease expression plasmid and an increase in NanoLuc luminescence is verified; and (ii) confirmation of reporter expression, in which firefly luciferase activity is measured and compared with that of non-transduced cells, because the firefly cassette is transcribed from the same transcript as the reporter. If independent verification is required, stable integration of the provirus and transcription of the reporter can additionally be confirmed by genomic PCR, RT-qPCR, or sequencing.*

*4. Puromycin is used only during initial selection. Maintain stable cells in complete DMEM without puromycin thereafter.*

**Part 2: Protease overexpression assay**

**A. Seeding of HEK293T cells**

1. Maintain HEK293T cells in DMEM + 10% FBS + Pen/Strep at 37 °C with 5% CO_2_.

2. One day before transfection, seed at 7 × 10^4^ cells/well in a 24-well plate in 500 μL of complete DMEM.

3. Incubate overnight at 37 °C with 5% CO_2_.

4. Confirm that the cells are healthy (criteria as in Part 1, step A3) and approximately 70%–80% confluent on the day of transfection.

**B. Co-transfection of FlipNanoLuc reporter and protease expression plasmids**

1. For each well, add 0.5 μg of FlipNanoLuc reporter plasmid and 0.5 μg of protease expression plasmid (or empty vector for negative control) to 100 μL of Opti-MEM (total DNA: 1 μg; ratio = 1:1). Vortex briefly. The plasmid combinations for each assay are summarized in Tables 2 and 3; each row corresponds to one well condition.

2. Add 3 μL of 1 mg/mL PEI MAX (DNA:PEI = 1:3, w/w). Vortex briefly.

3. Incubate at room temperature (20–25 °C) for 15–20 min.

4. Add the transfection complex dropwise to each well, then gently rock the plate by hand (approximately 10 times in each of the forward-backward and side-to-side directions) to ensure even distribution.

5. Do not replace the medium. Return the plate to the incubator and culture the cells at 37 °C with 5% CO_2_ until harvest.

*Note: In contrast with the SARS-CoV-2 infection protocol, medium replacement is not required in this assay.*

**C. Cell lysis**

Harvest time points:

a. 3CLpro overexpression: 48 h post-transfection (acceptable range 45–48 h).

b. Caspase-3 overexpression: 24 h post-transfection (acceptable range 20–24 h).

1. Aspirate the culture medium from each well.

2. Add 100 μL of 1× lysis buffer directly to each well.

3. Lyse immediately by pipetting up and down several times.

4. Use immediately or keep on ice for no more than 10 min.

*Notes:*

*1. Harvest times differ between the 3CLpro (48 h) and Caspase-3 (24 h) assays. Prolonged caspase-3 overexpression may induce cell death and increase background. These times are upper limits; harvesting later than 48 h (3CLpro) or 24 h (caspase-3) is not recommended.*

*2. Avoid generating bubbles during pipetting.*

**D. Luminescence measurement and normalization**

1. Measure NanoLuc and firefly luciferase activities in the lysates as described in Part 6.

2. Calculate the normalized activity and the fold induction as described in Part 7.

**Part 3: Apoptosis detection assay using staurosporine**

**A. Seeding of HEK293T cells stably expressing Casp3A-FlipNanoLuc**

1. Maintain HEK293T cells stably expressing Casp3A-FlipNanoLuc (see Table 1) in complete DMEM (DMEM + 10% FBS + Pen/Strep) at 37 °C with 5% CO_2_.

2. One day before drug treatment, seed at 7 × 10^4^ cells/well in a 24-well plate in 500 μL of complete DMEM.

3. Incubate overnight at 37 °C with 5% CO_2_.

4. Confirm that the cells are healthy (criteria as in Part 1, step A3) and approximately 70%–80% confluent on the day of treatment.

**B. Staurosporine treatment**

1. Prepare a 200 μM stock solution of staurosporine in DMSO. Dilute to a 1 μM working solution in complete DMEM immediately before use.

2. Prepare the DMSO vehicle control by diluting an equivalent volume of DMSO in complete DMEM (final DMSO concentration matching that of the staurosporine working solution).

3. Aspirate the culture medium from each well.

4. Add 500 μL of the appropriate treatment solution to each well:

a. 0-h control: DMSO vehicle control (harvest immediately after addition; see step B5).

b. 6 h: 1 μM staurosporine in complete DMEM.

5. For 0-h control wells, proceed immediately to Part 3, section C.

6. For 6-h treatment wells, incubate at 37 °C with 5% CO_2_ for 6 h, then proceed to Part 3, section C.

*Notes:*

*1. Staurosporine is a broad-spectrum kinase inhibitor and apoptosis inducer. Handle with appropriate care; avoid skin contact. Prepare working solutions fresh on the day of use.*

*2. DMSO concentration in the vehicle control must match that in the staurosporine working solution to exclude solvent effects on luminescence.*

*3. Visible cell rounding and detachment may be observed in staurosporine-treated wells at 6 h. This is expected and reflects the induction of apoptosis. The extent of detachment need not be quantified, because the detached cells are collected at lysis (Part 3, section C); do not discard the culture medium at any point. Judge successful induction from the fold induction of the normalized NanoLuc activity relative to the DMSO 0-h control, not from the morphology.*

**C. Cell lysis**

1. At each harvest time point (0 and 6 h), transfer the entire culture medium from each well to a 1.5 mL microcentrifuge tube.

2. Centrifuge at 500× *g* for 5 min at 4 °C.

3. Aspirate the supernatant to approximately 50 μL, then remove the remainder completely with a pipette. The pellet is small and may not be visible; do not disturb it.

4. Add 100 μL of 1× lysis buffer to the emptied well and lyse the adherent cells immediately by pipetting up and down several times.

5. Transfer the lysate to the corresponding tube and resuspend the pellet by pipetting up and down several times.

6. Use immediately or keep on ice for no more than 10 min.

*Notes:*

*1. Process every well identically, including the DMSO 0-h control wells, even when no detachment is visible. The detached cells are the population in which caspase-3 has been activated; discarding them from some wells underestimates the fold induction, and the firefly normalization does not correct this bias.*

*2. Resuspending the pellet in the lysate prepared from the same well keeps the final volume at 100 μL for all the samples, thereby avoiding the dilution of the lysis buffer by the carryover of the culture medium.*

*3. Luciferase released into the medium by secondary necrosis is not recovered at 500× g. This contribution is limited at 6 h, but increases with a longer treatment; therefore, do not extend the staurosporine treatment beyond 6 h.*

*4. Avoid generating bubbles during pipetting.*

*5. Fold induction is calculated relative to the mean of the DMSO 0-h control (set to 1.0); see Part 7.*

**D. Luminescence measurement and normalization**

1. Measure NanoLuc and firefly luciferase activities in the lysates as described in Part 6.

2. Calculate the normalized activity and the fold induction as described in Part 7.

**Part 4: OC43 coronavirus infection detection assay**

**A. Seeding of HEK293T cells stably expressing 3CLpro-SL1-FlipNanoLuc**

1. Maintain HEK293T cells stably expressing 3CLpro-SL1-FlipNanoLuc (see Table 1) in complete DMEM (DMEM + 10% FBS + Pen/Strep) at 37 °C with 5% CO_2_.

2. One day before infection, seed at 3 × 10^4^ cells/well in a 96-well plate in 100 μL of complete DMEM.

3. Incubate overnight at 37 °C with 5% CO_2_.

4. Confirm that the cells are healthy (criteria as in Part 1, step A3) and approximately 70%–80% confluent on the day of infection.

**B. OC43 infection**

1. Prepare OC43 inoculum at an MOI of 1 in complete DMEM. Adjust the volume so that 100 μL of inoculum is added per well.

2. Aspirate the culture medium from each well.

3. Add 100 μL of inoculum directly to each well.

4. For mock controls, add 100 μL of virus-free complete DMEM.

5. Incubate at 37 °C with 5% CO_2_ for 48 h without medium replacement.

*Note: MOI is calculated on the basis of the cell number at the time of seeding. The titer of the virus stock is determined in-house by a focus-forming assay before use; the titer stated by the supplier is not used for this calculation because the infectious titer depends on the cell line and the assay conditions.*

**Biosafety notice:** OC43 (human coronavirus OC43) is a BSL-2 pathogen. All experiments must be conducted in a certified BSL-2 facility in accordance with the institutional biosafety protocols and applicable regulations.

*Note: In contrast with the SARS-CoV-2 protocol (Part 5), no adsorption step or medium change is performed after virus addition in this assay.*

**C. Cell lysis**

1. At 48 h post-infection, aspirate the culture medium.

2. Add 50 μL of 1× lysis buffer directly to each well.

3. Lyse immediately by pipetting up and down several times.

4. Use immediately or keep on ice for no more than 10 min.

*Notes:*

*1. Lysis volume is reduced to 50 μL to account for the smaller well volume of the 96-well format, while maintaining the lysate-to-reagent ratio used in Part 6.*

*2. Avoid generating bubbles during pipetting.*

**D. Luminescence measurement and normalization**

1. Measure NanoLuc and firefly luciferase activities in the lysates as described in Part 6.

2. Calculate the normalized activity and the fold induction as described in Part 7.

**Part 5: SARS-CoV-2 infection detection assay**

**A. Seeding BHK cells stably expressing hACE2**

1. Maintain BHK cells stably expressing hACE2 in MEM + 10% FBS + Pen/Strep + 3 μg/mL puromycin at 37 °C with 5% CO_2_.

2. One day before transfection, seed at 1.5 × 10^5^ cells/well in a 24-well plate in 500 μL of complete MEM.

3. Incubate overnight at 37 °C with 5% CO_2_.

4. Confirm that the cells are healthy (criteria as in Part 1, step A3) and approximately 70%–80% confluent.

*Note: BHK cells stably expressing hACE2 are used because SARS-CoV-2 uses ACE2 as the principal host entry receptor. Parental BHK cells express little endogenous ACE2; therefore, ectopic expression of hACE2 renders these cells permissive to infection and makes viral entry strictly dependent on the introduced receptor (see General notes 2).*

**B. PEI-mediated transfection of FlipNanoLuc reporter plasmid**

1. Add 1 μg of 3CLpro-SL1-FlipNanoLuc reporter plasmid (see Table 1) to 100 μL of Opti-MEM. Vortex briefly.

2. Add 3 μL of 1 mg/mL PEI MAX (DNA:PEI = 1:3, w/w). Vortex briefly.

3. Incubate at room temperature (20–25 °C) for 15–20 min.

4. Add the transfection complex dropwise to each well, then gently rock the plate by hand (approximately 10 times in each of the forward-backward and side-to-side directions) to ensure even distribution.

5. Incubate at 37 °C with 5% CO_2_ for 24 h.

**C. SARS-CoV-2 infection**

**Biosafety notice:** SARS-CoV-2 is a BSL-3 pathogen. All experiments must be conducted in a certified BSL-3 facility under approved institutional biosafety protocols and applicable regulations.

1. At 24 h post-transfection, aspirate the transfection medium completely.

*Note: Thorough removal of the PEI-containing medium is critical; residual PEI inhibits SARS-CoV-2 attachment and markedly reduces reporter activation. If desired, wash once with plain MEM before adding the inoculum.*

2. Prepare SARS-CoV-2 inocula (MOI = 1, 3, 5, 10) in plain (serum-free) MEM, 100 μL per well.

3. Replace the medium by adding 100 μL of inoculum to each well (the well now contains the 100 μL inoculum only).

4. For mock controls, add 100 μL of virus-free plain MEM.

5. Incubate at 37 °C with 5% CO_2_ for 3 h to allow virus adsorption. Do not disturb the plate.

*Note: MOI is calculated on the basis of the cell number at the time of infection. Virus stock must be titrated using TCID_50_ prior to use.*

**D. Medium change after adsorption and infection incubation**

1. After the 3 h adsorption, carefully aspirate the inoculum.

2. Add 500 μL of complete MEM (MEM + 10% FBS) to each well.

3. Incubate at 37 °C with 5% CO_2_ for 24 h.

*Note: Recommended harvest time: Arakawa et al. [3] harvested at 48 h post-infection, whereas in this protocol, the cells are lysed after a 3-h adsorption followed by a 24-h incubation in complete MEM. The 24-h condition is recommended because robust reporter activation is reliably detected while cytopathic effects remain limited; extensive cytopathic effects at later time points are expected to compromise firefly normalization. The two harvest times were not systematically compared in this protocol.*

**E. Cell lysis**

1. After the 24-h incubation, aspirate the medium.

2. Add 100 μL of 1× lysis buffer directly to each well.

3. Lyse immediately by pipetting up and down several times.

4. Use immediately or keep on ice for no more than 10 min.

*Note: Avoid generating bubbles.*

**F. Luminescence measurement and normalization**

1. Measure NanoLuc and firefly luciferase activities in the lysates as described in Part 6.

2. Calculate the normalized activity and the fold induction as described in Part 7.

**Part 6: Measurement of NanoLuc and firefly luciferase activities (common procedure)**

1. Use a white 384-well plate.

2. Dispense samples into every other well (leave one empty well between occupied wells) to minimize optical crosstalk.

3. For each sample, prepare two separate wells: one for the Nano-Glo^®^ assay and the other for the Bright-Glo^TM^ assay.

4. Nano-Glo^®^ assay: Add 10 μL of lysate, then 10 μL of Nano-Glo^®^ reagent. Mix by pipetting three times.

5. Bright-Glo^TM^ assay: Add 10 μL of lysate, then 10 μL of Bright-Glo^TM^ reagent. Mix by pipetting three times.

6. Wait 2–5 min after reagent addition before measurement.

7. Measure with Varioskan LUX (or equivalent) using the following settings:

a. Plate type: white 384-well.

b. Mode: luminescence.

c. Reading: top.

d. Integration time: 1,000 ms.

e. Delay after reagent addition: 2–5 min.

f. Gain: default.

g. Sample volume: 10 μL/well

h. Reagent volume: 10 μL/well

*Notes:*

*1. Do not add both Nano-Glo^®^ and Bright-Glo^TM^ reagents to the same well. NanoLuc and firefly activities must be measured in separate wells from the same lysate.*

*2. Use identical instrument settings for all wells and all experiments.*

**Part 7: Normalization and data analysis (common procedure)**

1. Calculate normalized activity: Normalized activity = NanoLuc luminescence / Firefly luciferase luminescence.

2. Calculate fold induction: Fold induction = Normalized activity (experimental condition) / Normalized activity (control condition). The mean of the control condition is set to 1.0.

• Infection assays: mock-infected sample as control.

• Overexpression assays: empty vector co-transfection as control.

• Apoptosis assay: DMSO 0-h vehicle control as control.

3. Present data as mean ± SD of three biological replicates for each condition, that is, three independently seeded and treated (or independently infected) wells. In this protocol, biological replicates refer to independent wells processed in parallel within a single experiment, whereas technical replicates refer to repeated measurements of the same lysate. Because each lysate is measured once for NanoLuc and once for firefly luciferase activity, no technical replication is involved.

4. Repeat the entire experiment independently at least twice. The validation data presented in this protocol were obtained from two independent experiments that yielded consistent results. For quantitative comparisons, three or more independent experiments are recommended.

5. Statistical analysis: Compare two groups using a two-tailed Student's t-test, and compare three or more groups against a single control by one-way ANOVA followed by Dunnett's multiple comparison test, reporting multiplicity-adjusted P values. Examine the assumptions of these parametric tests using the residuals of the model, rather than the values of each group separately: assess normality (for example, by the Shapiro–Wilk test together with a QQ plot of the residuals) and equality of variances (for example, by the Brown–Forsythe test). Formal normality tests have limited power at small replicate numbers, so inspect the residual plots as well as the test results. If the variances are unequal but the residuals are approximately normal, use the Welch t-test for two groups or Welch's ANOVA for three or more, as these do not assume equal variances. Because fold induction is a ratio and varies multiplicatively rather than additively, the data may also be analyzed after logarithmic transformation, which compares geometric means and often improves both normality and homogeneity of variance; state whether the conclusion depends on the scale of analysis. If a rank-based test is preferred, use the Mann–Whitney U test for two groups or the Kruskal–Wallis test with Dunn's test for three or more, noting that these require larger replicate numbers to be informative: with two groups of three values, the smallest two-sided P value that the Mann–Whitney U test can return is 0.1. The statistical analyses reported in this protocol were performed using GraphPad Prism (version 11.0.0 for macOS).

*Note: Define the criteria for excluding a well before the data are examined. Exclude a well only for a documented technical reason, such as failure of reagent addition, contamination, unintended loss of the monolayer, or a response grossly discordant with that of the other replicate wells of the same condition. Cell rounding and detachment in the apoptosis assay (Part 3) and the cytopathic effect in the infection assays (Parts 4 and 5) are expected consequences of the treatment and are not, in themselves, grounds for exclusion. State any exclusion when data are reported.*

## General notes and troubleshooting

**General notes**

1. Plasmid availability: FlipNanoLuc reporter plasmids (including 3CLpro-FlipNanoLuc CoVA WT, 3CLpro-SL1-FlipNanoLuc CoVA WT, Q5A and SA, and Casp3A-FlipNanoLuc WT and D5A variants) are deposited at Addgene and are available on the Eiji Morita Lab depositor page (https://www.addgene.org/Eiji_Morita/). The Addgene ID for each construct is presented in Table 1 (see also [3]).

2. Generation of BHK cells stably expressing hACE2: These cells were generated as previously described [4]. Briefly, the hACE2 coding sequence was cloned into the PiggyBac transposon vector PB-CMV-MCS-EF1α-RedPuro (System Biosciences, catalog number: PB514B-2) and introduced into BHK cells (JCRB Cell Bank, JCRB9020) together with the Super PiggyBac transposase expression vector (System Biosciences, catalog number: PB200A-1; currently supplied as catalog number: PB210PA-1). Stable integrants were selected and maintained with 3 μg/mL puromycin in MEM supplemented with 10% FBS and Pen/Strep. Because this transposon vector co-expresses RFP together with the puromycin-resistance marker from the EF1α promoter, successful integration can also be monitored by RFP fluorescence. BHK cells were chosen because they express little endogenous ACE2; therefore, their susceptibility to SARS-CoV-2 depends on the introduced hACE2.

3. Retroviral vs. transient transfection: Stable cell lines (Parts 3 and 4) are preferred for infection assays because they ensure uniform reporter expression across all cells, thereby reducing well-to-well variability. Transient transfection (Parts 2 and 5) is sufficient for protease overexpression validation and SARS-CoV-2 detection.

4. Preparation of PEI MAX: Dissolve PEI MAX (MW 40,000) in water at 1 mg/mL. Filter-sterilize (0.22 μm), aliquot, and store at -20 °C. Thaw once and do not refreeze.

5. Firefly normalization: The firefly luciferase cassette is expressed from the same transcript as the FlipNanoLuc reporter via a P2A self-cleaving peptide and, therefore, serves as an internal control for transfection efficiency and cell number. No additional normalization plasmid is required.

6. Choice of cell lines: HEK293T cells are used both for retroviral packaging and as the reporter host because they are highly transfectable, support efficient production of retroviral particles, and are susceptible to human coronavirus OC43. BHK cells stably expressing hACE2 are used for the SARS-CoV-2 assay for the reason given in General note 2. The protocol was established using these immortalized cell lines and has not been tested in primary cells, in which transduction efficiency and reporter expression may be limited.

7. Adaptation to other proteases: Protease specificity is determined solely by the recognition sequence embedded in the flexible linkers; therefore, the reporter can, in principle, be redirected to another protease by replacing this sequence with a cognate cleavage motif spanning the P4–P1′ positions while keeping the linker length unchanged. In the present protocol, the system has been validated only for caspase-3 and coronavirus 3CLpro.

**Troubleshooting**

**Table:** 

Problem	Possible cause	Recommended solution
Low or no NanoLuc signal in all conditions	Insufficient cell lysis; reagent degradation	Ensure complete lysis by thorough pipetting. Check the expiry date of Nano-Glo^®^ reagent. Confirm that the reporter plasmid was successfully transfected/transduced.
High background NanoLuc in negative controls	Spontaneous β-strand refolding; protease leakage; contamination	Verify that cell lines are not mycoplasma-contaminated. Confirm that a negative control plasmid (C145A or D5A mutant) was used. Check for cross-contamination of virus stocks.
No fold induction after staurosporine treatment	Cells not responding to staurosporine; reporter cleavage site not matching active caspase-3	Confirm apoptosis induction by an independent assay (e.g., caspase-3 activity assay or Annexin V staining). Verify that the Casp3A reporter (not 3CLpro reporter) was used.
No signal increase after OC43 or SARS-CoV-2 infection	Residual PEI (SARS-CoV-2 only); low MOI; incorrect reporter; failed infection	For SARS-CoV-2: ensure complete medium change after transfection (Part 5, section C). Confirm MOI by TCID_50_. Verify infection by immunofluorescence or RT-PCR for viral antigen.
Inconsistent firefly signal between wells	Uneven cell seeding; pipetting error during lysis or reagent addition	Seed cells carefully to ensure uniform density. Use a multichannel pipette for reagent dispensing. Do not exclude wells solely on the basis of deviation from the group mean. Exclude a well only for a prespecified and documented technical reason.
High coefficient of variation (CV) between technical replicates	Bubbles in wells; optical crosstalk between adjacent wells	Avoid generating bubbles during lysis and reagent addition. Confirm every-other-well dispensing pattern. Allow adequate equilibration time (2–5 min) before reading.
Poor or variable transduction efficiency between preparations	Insufficient or variable retroviral input; loss of titer through freeze/thaw	Standardize the preparation of the retroviral supernatant (packaging cell number, plasmid amounts, harvest time) and the volume used for transduction, or determine the titer of each batch. Use fresh supernatant where possible and avoid repeated freeze/thaw cycles.
Low or variable NanoLuc signal in the stable population	Incomplete establishment of the stable population; insufficient reporter expression; incomplete selection	Confirm establishment of the population by protease-induced luminescence and by firefly luciferase activity relative to non-transduced cells. Where independent confirmation is needed, verify integration and expression by genomic PCR or RT-qPCR. Repeat the puromycin selection if the non-transduced control dish has not been fully eliminated.

## Validation of protocol

The FlipNanoLuc system and the experimental conditions described in this protocol were validated in the original study [3]. Key validation results are summarized below.

**Protease specificity (Part 2):** Co-expression of SARS-CoV-2 3CLpro (WT) with the cognate 3CLpro-FlipNanoLuc reporter yielded a >10-fold induction of normalized NanoLuc activity compared with empty vector controls. No significant induction was observed with the catalytically inactive C145A mutant protease or reporters bearing cleavage-site mutations (Q5A and SA), thereby confirming protease-dependent and sequence-specific activation.

**Caspase-3 detection (Part 2):** Overexpression of caspase-3 with the Casp3A-FlipNanoLuc reporter resulted in a significant induction of luminescence; no induction was observed with the D5A cleavage-site mutant reporter.

**Apoptosis detection (Part 3):** Treatment of HEK293T cells stably expressing Casp3A-FlipNanoLuc with 1 μM staurosporine for 6 h resulted in a significant increase in normalized NanoLuc activity relative to the DMSO 0-h control, consistent with caspase-3 activation during apoptosis.

**Figure 1.**
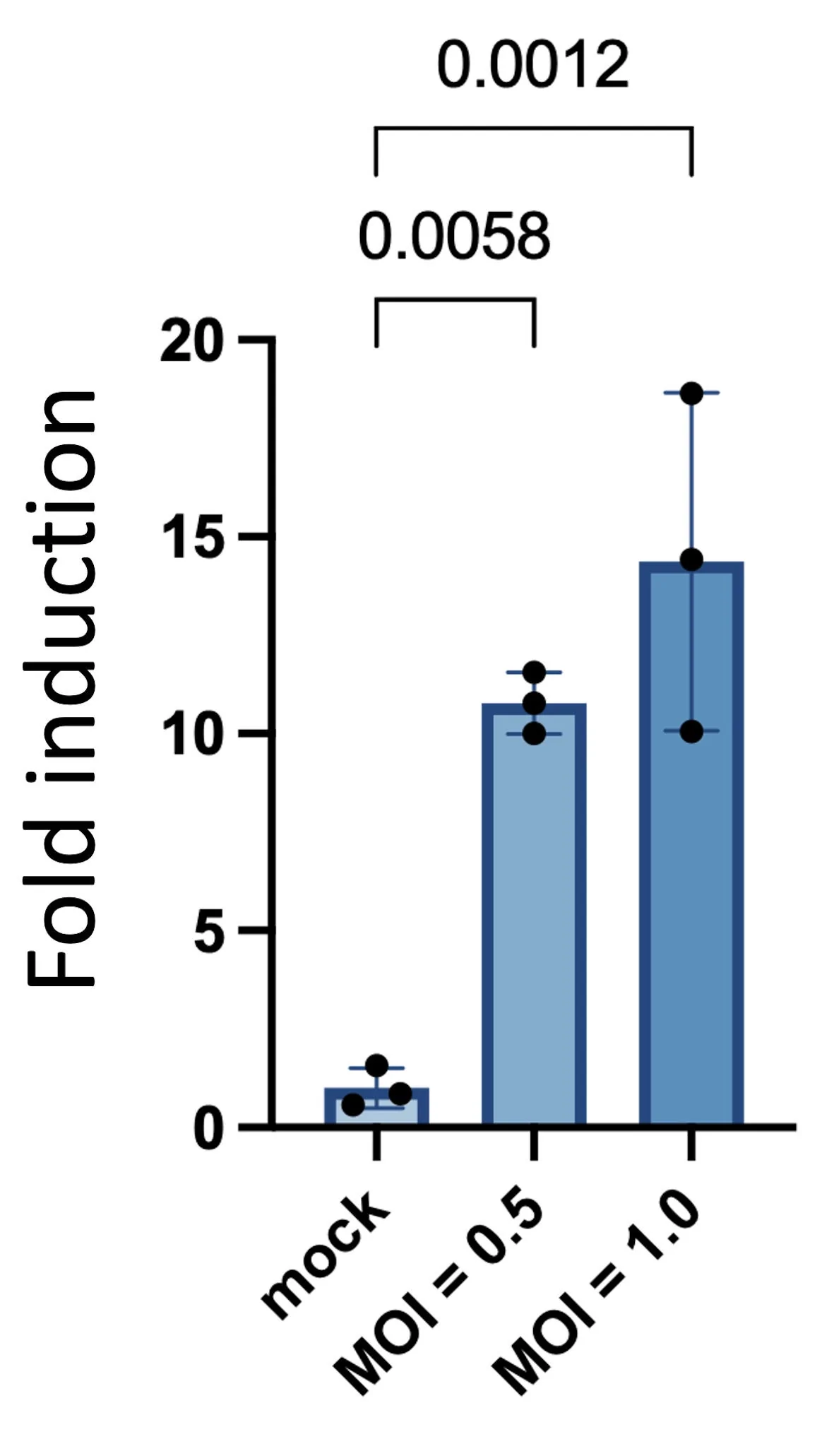
Detection of human coronavirus OC43 infection using the FlipNanoLuc protease biosensor. HEK293T cells stably expressing 3CLpro-SL1-FlipNanoLuc were infected with human coronavirus OC43 (HCoV-OC43) at an MOI of 0.5 or 1.0 and lysed at 72 h post-infection. Firefly and NanoLuc luciferase activities were measured, and NanoLuc activity was normalized to firefly luciferase activity. Data are expressed as the fold induction relative to the mean of the mock-infected control (set to 1.0) and presented as the mean ± SD of three independently infected wells within a single experiment. The experiment shown is representative of two independent experiments that gave consistent results. Multiplicity-adjusted P values from one-way ANOVA followed by Dunnett's multiple comparison test vs. the mock-infected control are shown in the figure. Analysis of log10-transformed values gave the same conclusion (adjusted P = 0.0002 and P = 0.0001 for MOI 0.5 and 1.0, respectively).

**OC43 infection detection (Part 4):** Infection of HEK293T cells stably expressing 3CLpro-SL1-FlipNanoLuc with OC43 at an MOI of 1 produced significant reporter activation at 48 h post-infection compared with mock-infected cells, demonstrating the detection of 3CLpro activity during authentic coronavirus infection. As additional representative validation data, HEK293T cells stably expressing 3CLpro-SL1-FlipNanoLuc infected with OC43 at an MOI of 0.5 or 1.0 showed a dose-dependent increase in normalized NanoLuc activity at 72 h post-infection (Figure 1).

**SARS-CoV-2 infection detection (Part 5):** Transient transfection of 3CLpro-SL1-FlipNanoLuc into BHK cells stably expressing hACE2, followed by SARS-CoV-2 infection (MOI = 1–10), resulted in a dose-dependent increase in the normalized NanoLuc signal at 24 h post-infection, thereby confirming reporter sensitivity to authentic SARS-CoV-2 3CLpro activity.
